# Medical Items

**Published:** 1893-11

**Authors:** 


### *MEDICAL ITEMS.*

THE poem which appeared in our last issue, kindly sent us by our friend, Dr. C. W. Thompkins, of Jasper, Florida, was incorrectly attributed to the Jasper *News*. It first appeared in the columns of the Mayo *Free Press*, and was written by Dr. Robt. Lee Goodbred, of Mayo, Florida.

---

SIR ASTLEY COOPER used to address every candidate for membership in the Royal College of Surgeons of England, of which he was president, somewhat in these words: "Gentlemen, you are about to enter on a noble and difficult profession; your success in it will depend on three things: first, a good and thorough knowledge of your profession; second, an industrious discharge of its duties; and third, the preservation of your moral character. Without the first no one can wish you to succeed; without the second you cannot succeed; and without the third, even if you do succeed, success can bring you no happiness."—*Ernest Hart.*

---

*Polk's Medical Register of the United States for 1893* contains the names of more than 105,000 practitioners, including "paths" of every variety. Of the 137 lawfully established colleges in the United States and Canada, 23 now require four courses of instruction of not less than six months each; 23 require four or more years of study and three courses of not less than six months each; 55 require three or more years of study and three terms of not less than six months each. Of the remaining 36, all but 7 have announced a minimum requirement of three courses of instruction before candidates are admitted to the final examination. Nearly every medical college in the United States now requires clinical instruction, laboratory instruction in chemistry, toxicology, the analysis of urine, normal and pathological histology, bacteriology, pathology, and hygiene. More than ninety per cent, of the regular colleges in the United States now require preliminary education equal to or better than that required in high schools of the first class.—*Medical Record.*

---

MR. W. B. SAUNDERS, publisher, of Philadelphia, Pa., announces the American Text-book of Gynecology as ready for early issue. It is the joint work of Drs. Howard Kelley, Pryor, Byford, Baldy, Tuttle and others who stand before the profession for all that is progressive in gynecology. The work will contain operations not before described in any other book, notably ablation of fibroid uterus. It is designed as a profusely illustrated reference book for the practitioner, and every practical detail of treatment is precisely stated.

---

THE College of Physicians of Philadelphia announces that the next award of the Alvarenga Prize, being the income for one year of the bequest of the late Señor Alvarenga, and amounting to about one hundred and eighty dollars, will be on July 14, 1894, provided that an essay deemed by the Committee of Award to be worthy of the prize shall have been offered.

Essays intended for competition may be upon any subject in medicine, but cannot have been published, and must be received by the Secretary of the College, Dr. Charles W. Dulles, on or before May 1, 1894.

Each essay must be sent without signature, but must be plainly marked with a motto and be accompanied by a sealed envelope having on its outside the motto of the paper and within the name and address of the author.

It is a condition of competition that the successful essay or a copy of it shall remain in possession of the College; other essays will be returned upon application within three months after the award.

---

THE United States grand jury at Chattanooga, Tennessee, has ignored the bill in the criminal suit for libel against Dr. James E. Reeves for his denunciation of the so-called "Amick Cure for Consumption" as quackery and fraud. There is still a civil suit pending, but Dr. Reeves is afraid that it will not be pressed, as the rules of civil suits will permit him to introduce testimony in support of his statements which in criminal proceedings might have been fought out. He says that he welcomes the opportunity "to expose the vile thing," and that if he once gets before a court with his testimony there will be nothing left of the fraud.

Dr. Reeves is to be congratulated for a display of courage in standing up for the right, which is unfortunately rare among medical men to-day. The number of so-called medical journals that have given advertising and reading columns to the exploitation of this "Amick" nostrum is a humiliating spectacle. The continued violation of the rule forbidding advertising of nostrums by the trustees of the *Journal of the American Medical Association* is another sight that should bring the blush of shame to the cheeks of every member of the great association defiantly misrepresented by its elected officers. To think that the absence of one man from the Milwaukee meeting caused the matter to be passed over in silence is an evidence of general pusillanimity that makes courage like that of Dr. Reeves stand out in bold relief.—*Medical News.*

A private letter from Dr. Reeves states that "we have the quack Amick on the run, and we claim it another victory for the medical profession of the South." We heartily congratulate Dr. Reeves upon his success in this fight.

